# Multisystem inflammatory syndrome in children related to COVID-19: Data from a Mexican national referral children's hospital

**DOI:** 10.3389/fped.2022.949965

**Published:** 2022-08-12

**Authors:** Héctor Menchaca-Aguayo, Deshire Alpizar-Rodriguez, Pamela Ramos-Tiñini, Enrique Faugier-Fuentes

**Affiliations:** ^1^Pediatric Rheumatology Department, Hospital Infantil de México Federico Gómez, Mexico City, Mexico; ^2^Research Unit, Colegio Mexicano de Reumatología, Mexico City, Mexico

**Keywords:** pediatric inflammatory multisystem syndrome temporally associated with COVID-19, PIMS-TS, Multisystem Inflammatory Syndrome in Children (MIS-C), SARS-CoV-2, COVID-19, epidemiology, pediatrics

## Abstract

**Objectives:**

To describe characteristics of patients with the pediatric inflammatory multisystem syndrome, temporally associated with SARS-CoV-2 (PIMS-TS)/multisystem inflammatory syndrome in children (MIS-C) and to identify factors associated with admission to the pediatric intensive care unit (PICU) in the Mexican children without coronavirus disease 2019 (COVID-19) vaccination.

**Methods:**

This was a cross-sectional study performed at Hospital Infantil de Mexico Federico Gomez, a referral children's hospital in Mexico. The study included all cases that met the criteria for PIMS-TS/MIS-C, unvaccinated, between March 2020 and January 2022. The primary outcome was the admission to PICU. Associations of PICU admission with demographic and clinical variables were estimated using logistic regression analyses.

**Results:**

We identified a total of 90 cases, with a median age of 7.5 years old, 47 (52.2%) girls. A previously healthy status was recorded in 76 (85%) children. All patients had positive PCR, serology test, or COVID-19 exposure. PICU admission was reported in 41 (45.6%) children. No deaths were reported. Patients received as treatment only corticosteroids in 53.3% of the cases. In univariable analyses, baseline factors associated with PICU admission were older age, hypotension or shock, positive PCR test, hypoalbuminemia, elevated procalcitonin, ferritin, and lymphopenia. Age, shock at admission, and hypoalbuminemia remained independently associated in the multivariable analysis adjusted by gender and previously healthy status.

**Conclusion:**

We found a high proportion of previously healthy children in patients with PIMS-TS/MIS-C in our center. Critical care attention was received by nearly half of the children. The main treatment used was steroids. Age, shock at admission, and hypoalbuminemia were factors associated with PICU admission.

## Introduction

In December 2019, severe acute respiratory syndrome coronavirus 2 (SARS-CoV-2) was described as the causal agent of coronavirus disease 2019 (COVID-19) (1). Early in the pandemic, the main manifestations in children with COVID-19 were fever, cough, and mild pneumonia, and the epidemiology was obscured due to many asymptomatic cases (1). However, a severe multisystem inflammatory syndrome was described in children under 21 years old in Europe and the United States. This syndrome had similar features to Kawasaki disease and toxic shock syndrome (2). After noticing a gradual increase in cases of children with multisystem inflammation as a late response to SARS-CoV-2 infection, the definition of pediatric inflammatory multisystem syndrome temporally associated with SARS-CoV-2 (PIMS-TS) was developed by The Royal College of Pediatrics and Child Health (RCPCH) and Multisystem Inflammatory Syndrome in Children (MIS-C) by the World Health Organization (WHO) and the Centers for Disease Control and Prevention (CDC) (3).

At the beginning of the pandemic, the incidence of PIMS-TS/MIS-C was reported as 2 per 100,000 individuals younger than 21 years of age (4). As of March 2022, the CDC in the United States reported a total of 7,459 cases and 63 deaths (5). In a cohort study, the incidence was 5.1 cases per million persons-months and 316 per million persons with SAR-CoV-2 younger than 21 years old (6). In the latest epidemiological update of COVID-19 of the z(PAHO) and WHO on December 2nd, 2021, a total of 8,686 cumulative confirmed cases of PIMS-TS/MIS-C were reported, including 165 deaths with a fatality rate of 1.9% (7). In Mexico, as of March 2022, a total of 434,238 confirmed cases of COVID-19 and 1,078 deaths in children under 19 years of age were reported (8). However, there is currently no official report on the number of cases of PIMS-TS/MIS-C in the Mexican population.

Since the emergence of this new entity, a wide clinical spectrum of clinical signs and symptoms have been described, characterized by persistent fever and multisystem involvement, predominantly gastrointestinal, cardiac manifestations, and shock status (9, 10). Different clinical phenotypes have been described in order to standardize both diagnosis and treatment: Kawasaki disease-like presentation, undefined inflammatory presentation, and shock-like presentation (9). A significant proportion of patients experienced severe disease requiring admission to the Pediatric Intensive Care Unit (PICU) (11–13). Immunomodulatory therapies have been used to reverse the hyperinflammatory state, mainly immunoglobulin and corticosteroids (14, 15). In cases of refractoriness, biologics such as anakinra, infliximab, and tocilizumab have been used (16, 17). Anticoagulation and platelet antiaggregating treatment are described as adjuvant therapy (16). However, the response to treatment may vary due to genetic susceptibility (18). It is interesting to observe the clinical behavior in the context of the emergence of new SARS-CoV-2 variants and during the different pandemic waves. Considering this entity's relevance, it is necessary to carry out studies that contribute to the national and international epidemiology. The results of research studies may allow to develop guidelines focused on timely diagnosis and adequate treatment, in order to prevent complications that can lead to fatal outcomes in patients.

The aim of this study was to describe the clinical characteristics of patients with PIMS-TS/MIS-C and to identify the factors associated with admission to the PICU in a third-level hospital in Mexican children without COVID-19 vaccination.

## Patients and methods

### Patients and study outcomes

This was a cross-sectional study performed at Hospital Infantil de Mexico Federico Gomez, a national referral children's hospital in Mexico City. The study included all consecutive patients younger than 18 years old that met the criteria for PIMS-TS/MIS-C of the RCPCH, WHO, and/or CDC, who were hospitalized between March 2020 and January 2022. All patients were unvaccinated against SARS-CoV-2. Medical records were reviewed at the end of the hospitalization of each patient. The protocol was approved by the local ethics committee (Project Number HIM-2021-018). Since this was a study comprising a review of de-identified data, written informed consent was not required.

Variables assessed in the analysis were demographic data, clinical and laboratory data, such as age, sex, and clinical variables, including hospitalization days, symptoms duration, diagnosis at admission and all components of clinical criteria of WHO and CDC and treatment. We further analyzed levels of inflammatory markers, such as erythrocyte sedimentation rate (ESR), C-reactive protein (CRP), procalcitonin, and D-dimer. We included only patients with exposure to a suspected or confirmed COVID-19 cases within 4 weeks prior to the onset of symptoms. Treatment received during hospitalization was recorded. The primary outcome on this analysis was the admission to the intensive care unit (PICU).

The number of PIMS-TS/MIS-C cases registered by month at our center was graphically compared to the total number of COVID-19 cases by month in the Mexican general population reported by WHO throughout the pandemic (19).

### Statistical analysis

Characteristics were examined for their association with admission to PICU using descriptive statistics; chi-squared or Fisher's exact test for categorical variables, *T*-student or Mann–Whitney *U*-test for continuous variables. In addition, we computed odds ratios (*OR*) with 95% confidence intervals (*CI*) to examine the association between admission to PICU and covariates, using logistic regression analyses. We analyzed univariable and multivariable associations by adjusting for potential confounders, such as age, gender, and previously healthy status. The *p*-values < 0.05 were considered statistically significant. All analyses were performed with STATA 14.0 (Stata Corp LP, College Station, TX, USA).

## Results

We identified a total of 90 cases. Children had a median age of 7.5 years old (interquartile range, IQR 2–11), the youngest being 2 months old and the oldest 17 years old, with 47 (52.2%) being female. Most of the children were previously healthy, 76 (85.4%).

Of all the above-mentioned cases, 41 (45.6%) were admitted to PICU, and 22 (24.4%) required mechanical ventilation. There was one documented case of macrophage activation syndrome (MAS), but no cases of disseminated intravascular coagulation (DIC) or deaths were reported.

Table 1 describes the demographic and clinical characteristics as well as the laboratory data at baseline in patients with PIMS-TS/MIS-C in children in our center by PICU admission status, from March 2020 to January 2022 of our studied population. No differences were noted based on sex. All of the patients had a history of positive PCR, serology test, or exposure to COVID-19.

**Table 1:** Demographic, clinical characteristics, and laboratory data at baseline in patients with Multisystem Inflammatory Syndrome in Children in Hospital Infantil de México Federico Gomez by PICU admission status, from March 2020 to January 2022.

**Characteristics**	**Total** ***N* = 90**	**PICU** **41 (45.6%)**	**NO PICU** **49 (54.4%)**	**Univariable^a^** **OR (95% CI)**	***p***
Age, median (IQR)	7.5 (2–11)	**10.0 (7–12)**	3.0 (2–8)	**1.18 (1.1–1.3)**	**0.001**
Female sex, *n* (%)	47 (52.2)	20 (48.8)	27 (55.1)	0.7 (0.3–1.8)	0.550
Overweight and/or Obesity (WHO), *n* (%)^b^	25 (31.7)	13 (38.2)	12 (26.7)	1.7 (0.7–4.4)	0.276
Previously healthy, *n* (%)	76 (85.4)	34 (82.9)	42 (87.5)	0.7 (0.2–2.3)	0.544
Hospitalization days, median (IQR)	6.0 (4–9)	7.0 (5–11)	5.0 (3–7)	1.1 (0.9–1.2)	0.087
Symptoms duration days, median (IQR)	6.0 (4–9)	6 (3–8)	7 (6–10)	0.9 (0.9–1.0)	0.092
Number of previous medical visits due to current illness	3.0 (2–4)	3 (2–4)	3 (2–4)	1.3 (0.9–1.9)	0.104
Kawasaki diagnosis at admission	19 (21.1)	6 (14.6)	13 (26.5)	0.5 (0.2–1.4)	0.174
PIMS diagnosis at admission	75 (83.3)	34 (82.9)	41(83.7)	1.1 (0.3–4.3)	0.908
RT-PCR antigen test for COVID-19	32 (36.4)	**21 (52.5)**	11 (22.9)	**3.7 (1.5–9.3)**	**0.005**
Positive serology for COVID-19^c^	19 (67.9)	11 (78.6)	8 (57.1)	2.8 (0.5–14.4)	0.232
Contact with patients with COVID-19 (4 weeks before)	74 (84.01)	30 (73.2)	44 (93.6)	0.2 (0.1–0.8)	0.185
Positive test or contact	90 (100)	41 (100)	49 (100)	-	-
**WHO PIMS criteria**, ***n*** **(%)**
Age 0–19 years	90 (100)	41 (100)	49 (100)	-	-
Fever >3 days	72 (80.0)	31 (75.6)	41 (83.7)	0.6 (0.2–1.7)	0.343
Rash or bilateral non-purulent conjunctivitis or mucoutaneous inflammation signs	76 (84.4)	34 (82.9)	42 (85.7)	0.8 (0.3–2.5)	0.717
Hypotension or shock	41 (45.6)	**33 (80.5)**	8 (16.3)	**21.1 (7.2–62.4)**	**<0.001**
Features of myocardial dysfunction, pericarditis, valvulitis or coronary abnormalities	74 (82.2)	35 (85.4)	39 (79.6)	1.5 (0.5–4.5)	0.477
Elevated D dimer > 550 ng/ml	86 (95.6)	39 (95.1)	47 (95.2)	0.8 (0.1–6.2)	0.855
Acute gastrointestinal problems	70 (77.8)	33 (80.5)	37 (75.5)	1.3 (0.5–3.7)	0.572
Elevated markers of inflammation [ESR (>10), procalcitonin (>2), CRP (>0.3)]	88 (97.8)	40 (97.6)	48 (97.9)	0.8 (0.1–13.7)	0.899
Negative hemocultures^d^	80 (100)	38 (100)	42 (100)	-	-
**CDC PIMS criteria**, ***n*** **(%)**
Age < 21 years	90 (100)	41 (100)	49 (100)	-	-
Fever > 38 ≥ 24 h	90 (100)	41 (100)	49 (100)	-	-
Elevated CRP^e^	74 (96.1)	32 (96.9)	42 (95.4)	1.5 (0.1–17.5)	0.736
Elevated ESR^e^	63 (84.0)	28 (84.8)	35 (83.3)	1.1 (0.3–3.9)	0.859
Elevated fibrinogen	63 (73.3)	28 (70.0)	35 (76.1)	0.7 (0.3–1.9)	0.525
Elevated procalcitonin^f^	20 (36.4)	**15 (50.0)**	5 (20.0)	**4.0 (1.2–13.4)**	**0.025**
Elevated ferritin^g^	34 (57.6)	**22 (73.3)**	12 (41.4)	**3.9 (1.3–11.7)**	**0.015**
Elevated HDL^g^	43 (70.5)	17 (60.7)	26 (78.8)	0.4 (0.1–1.3)	0.128
Lymphopenia	44 (49.4)	**28 (68.3)**	16 (33.3)	**4.3 (1.8–10.5)**	**0.001**
Hypoalbuminemia^b^	68 (77.3)	**36 (90.0)**	32 (66.7)	**4.5 (1.4–14.9)**	**0.014**
Glasgow Coma Scale, mean (SD)	14.8 (0.6)	14.7 (0.8)	14.9 (0.2)	0.3 (0.1–1.1)	0.06

^a^Logistic regression analysis.

^b^n = 79.

^c^n = 28.

^d^n = 80.

^e^n = 77.

^f^n = 55.

^g^n = 59.

IQR, interquartil range 25–75.

Bold values indicate statistically significant results.

Factors preceding admission significantly associated with the admission to PICU were age, *OR* 1.18 (95% *CI*: 1.1–1.3); hypotension or shock at admission, *OR* 21.1 (95% CI: 7.2–62.4); positive RT-PCR antigen test for COVID-19, *OR* 3.7 (95% *CI*: 1.5–9.3); CRP ≥ 7.4 (median in our population), *OR* 5.2 (95% *CI*: 1.9–13.8); elevated procalcitonin and ferritin (*OR* 4.0, 95% *CI*: 1.2–13.4 and *OR* 3.9, 1.3–11.7, respectively); lymphopenia, *OR* 4.3 (95% *CI*: 1.8–10.5) and hypoalbuminemia *OR* 4.5 (95% *CI* 1.4–14.9). In the multivariable analysis adjusted by gender and previously healthy status, age (*OR* 1.2, 95% *CI*: 1.1–1.5) and hypoalbuminemia (*OR* 13.4, 95% *CI*: 2.4–75.5), shock at admission (*OR* 23.7, 95% *CI*: 6.1–92.2) remained significantly associated with PICU admission.

Table 2 shows the management of 90 patients with PIMS-TS/MIS-C in Children in our center by PICU admission status from March 2020 to January 2022. In 53.3% of cases, only corticosteroids were used and the combination of intravenous immunoglobulin (IVIG) and corticosteroids was utilized in 40.0%. No children received extracorporeal membrane oxygenation (ECMO).

**Table 2:** Management of 90 patients with Multisystem Inflammatory Syndrome in Children in Hospital Infantil de México Federico Gomez, by PICU admission status from March 2020 to January 2022.

**Treatment, *n* (%)**	**Total** ***N* = 90**	**PICU** **41 (45.6%)**	**NO PICU** **49 (54.4%)**	**Univariable** **(95% IC)**	***P***
Only corticosteroids	48 (53.3)	25 (60.9)	23 (46.9)	1.8 (0.8–4.1)	0.185
IVIG + corticosteroid	36 (40.0)	15 (36.6)	21 (42.9)	0.8 (0.3–1.8)	0.546
Vasopresors	37 (41.1)	36 (87.8)	1 (2.0)	**345.6 (38.7–3,088.3)**	**<0.001**
Biologic treatment (Infliximab)	2 (2.2)	1 (2.4)	1 (2.0)	1.2 (0.1–19.8)	0.899
Plasmapheresis	1 (1.1)	1 (2.4)	0	-	-
Replacement therapy	1 (1.1)	1 (2.4)	0	-	-
ECMO	0	0	0	-	-

Bold values indicate statistically significant results.

**Figure 1:**
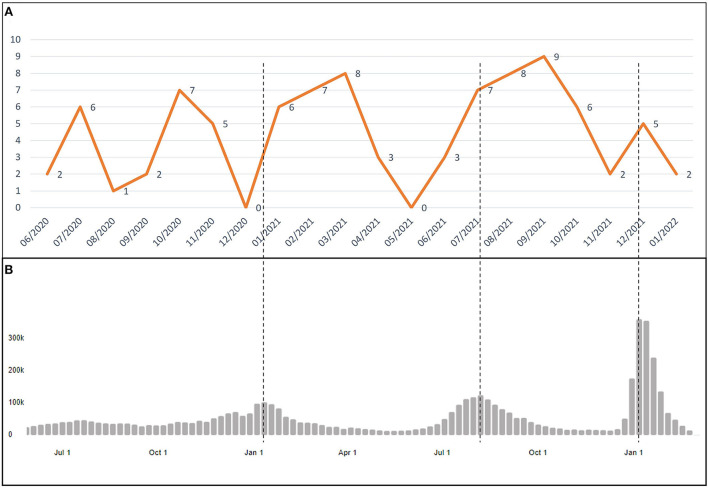
Temporal distribution of pediatric inflammatory multisystem syndrome, temporally associated with SARS-CoV-2 (PIMS-TS)/multisystem inflammatory syndrome in children (MIS-C) cases from March 2020 to January 2022. **(A)** Number of cases of PIMS-TS/MIS-C in “Hospital Infantil de Mexico Federico Gomez” by month and **(B)** COVID-19 cases in general population in Mexico by month reported by WHO.

Figure 1 displays the number of PIMS-TS/MIS-C cases and the total number of COVID-19 cases in the Mexican general population by month reported by WHO (19). The first patient was registered in our database in June 2020. An increase in the number of cases of PIMS-TS/MIS-C after the peak of waves of COVID-19 in the general population in Mexico was observed.

## Discussion

The present study focused on the description of characteristics of patients with PIMS-TS/MIS-C in a national referral center in Mexico and identification of factors associated with admission to PICU. The highest prevalence was observed in previously healthy school-aged children. This finding is in line with previous reports in the literature where MIS-C was described as predominantly affecting children between 6 and 12 years of age (20). The age range of presentation has been reported from 7 months to 20 years (21). The previous studies suggested a greater predilection for male gender; however, later studies did not reveal broad differences in this variable (11, 21, 22).

The most frequent manifestations in our study were fever, mucocutaneous, gastrointestinal, and cardiovascular. These results are similar to those reported by different cohorts during the pandemic's course. In a meta-analysis including 27 studies with a large population of 917 patients, the most common symptoms were fever and gastrointestinal involvement (22). Another systematic review published by Hoste et al. reported that fever was documented in all patients during the first 5 days; 85.6% presented gastrointestinal symptoms such as diarrhea, abdominal pain, and vomiting; followed by cardiovascular disease in 79.3% (11).

Regarding laboratory findings, D-dimer elevation was reported in 97% of patients, and in all patients, inflammatory markers were considered within the aforementioned definition. These are related to the secretion of multiple inflammatory cytokines (20). Other biomarkers, such as ferritin, fibrinogen, and cardiac enzymes, have also been described (21). The elevation of procalcitonin and pro-BNP has been associated with admission to the PICU (13, 23). The first cases of PIMS-TS/MIS-C were described 4 weeks after acute SARS-CoV-2 infection (3). To complement the diagnosis, study tests such as PCR for SARS-CoV-2 and serology had been used. Approximately 75% of the children in the different published cut-offs had antibodies for SARS-CoV-2; and 52% had positive PCR (20). In our study, 36% had positive PCR tests for SARS-CoV-2 on admission. We found that PCR for SARS-CoV-2 was frequently positive in patients admitted to the PICU.

In a systematic review, a severe course of the disease was reported in up to 86% of patients. This was related to older age, gastrointestinal, and cardiovascular symptoms (11). A recent study found that the presence of anemia, diarrhea, hypoxia, altered mental status, and seizures or shock were risk factors for PICU admission (24). In our study, the associated factors were age, hypoalbuminemia, and shock at admission.

During the course of the pandemic, different clinical behaviors have been described, being denominated presentation phenotypes of MIS-C. In the MIS-C management guidelines in Switzerland, these phenotypes were classified in the following presentations: shock, a presentation similar to Kawasaki disease and indefinite inflammatory (9). This classification was used for our analysis; from our patients, 46% had shock presentation, 21% had a presentation similar to Kawasaki disease, and the remaining had indefinite inflammatory presentation. Other publications describe presentations with characteristics similar to Kawasaki disease in 40% (25).

Regarding the treatment used, most of the patients in our study were treated only with corticosteroids with favorable results (26). Infliximab was used in two refractory cases with presentations similar to Kawasaki disease (26). Among the comorbidities presented in our study, the relationship between overweight and obesity stands out in 31.7% (20). Other systematic reviews have found obesity as an associated comorbidity (11). Most of the patients in our study had no comorbidities.

We compared the number of PIMS-TS/MIS-C cases by month in our center and the total number of COVID-19 cases in the Mexican general population reported by WHO (19). We observed an increase in the number of cases of PIMS-TS/MIS-C after the peak of waves of COVID-19, which could be explained because PIMS-TS/MIS-C is a late immunology response after an acute SARS-CoV-2 infection.

Currently, with the spread of new variants among non-vaccinated pediatric populations, the spectrum of manifestations in children has changed. Among the last cases included for this analysis, we observed a higher incidence of similar to Kawasaki disease phenotype, compared to shock presentation.

A limitation of our study was the cross-sectional design. This was a single-center study. The small sample size has limited statistical power. However, this is one of the largest reports from a Latin American center and is the first report of PIMS-TS/MIS-C in exclusively Mexican population throughout the pandemic. Another strength of our study is the use of widely accepted definitions.

## Conclusion

The present study depicts the experience of our institution with PIMS-TS/MIS-C. Our population was predominantly healthy without significant comorbidities. Nearly half of children received care in PICU, the associated factors were age, shock at admission, and hypoalbuminemia. The main treatment used was corticosteroids. We highlighted the null mortality.

## Data availability statement

The original contributions presented in the study are included in the article/supplementary material, further inquiries can be directed to the corresponding author.

## Author contributions

HM-A, PR-T, and EF-F performed data collection and data analysis. DA-R performed data analysis. EF-F conceptualized the study. All authors wrote and edited the manuscript and read and approved the final version of the manuscript.

## Conflict of interest

DA-R is a scientific advisor for GSK unrelated to this study. EF-F has been speaker for Abbvie, Roche, and Pfizer unrelated to this study. The remaining authors declare that the research was conducted in the absence of any commercial or financial relationships that could be construed as a potential conflict of interest.

## Publisher's note

All claims expressed in this article are solely those of the authors and do not necessarily represent those of their affiliated organizations, or those of the publisher, the editors and the reviewers. Any product that may be evaluated in this article, or claim that may be made by its manufacturer, is not guaranteed or endorsed by the publisher.
